# Watching movies in VR: A research ecosystem for the study of screen media effects

**DOI:** 10.3758/s13428-025-02750-y

**Published:** 2025-07-25

**Authors:** Faith A. Delle, Gary Bente, Nolan Jahn, Juncheng Wu

**Affiliations:** 1 Department of Advertising and Public Relations, Michigan State University, East Lansing, MI USA; 2 Department of Communication, Michigan State University, East Lansing, MI USA; 3 Institute of Advertising, Communication University of China, Beijing, China

**Keywords:** Virtual reality, Media effects, Emotions, Psychophysiology, Continuous response measurement

## Abstract

We introduce a virtual reality (VR) research ecosystem for the study of screen media effects and present a study providing evidence for its usability and validity. The study tested whether responses to affect-laden films presented on a standard TV within a physical space can be replicated in a virtual environment. The virtual setting was developed using Vizard 7.0, an open-access and customizable solution for media research. Using a between-subjects design, 70 participants were randomly assigned to either a TV or VR condition. Both groups were exposed to the same set of nine movie clips, encompassing three emotional categories (scary, funny, sad). While participants in the TV condition watched the clips on a physical 65″ TV, participants in the VR condition watched the clips on a virtual screen in the VR living room using a Meta Quest 2 VR headset. Continuous ratings of perceived emotional intensity and physiological measures of arousal (skin conductance level, heart rate, pulse volume amplitude) served as dependent variables. Overall, results confirmed the convergent validity between the two experimental conditions, revealing high correlations for all process variables across all stimuli. Results also demonstrated distinct responses to the clips of different emotional tones that were consistent across the experimental conditions. The findings encourage the use of VR for the study of screen media effects, demonstrating convergent validity with real-world scenarios while offering significant advantages, such as standardization and portability of the experimental setup as well as high levels of experimental control over reception setting variables.

**Supplementary Information:**

The online version contains supplementary material available at 10.3758/s13428-025-02750-y.

## Introduction

Virtual reality (VR) technology is expected to dramatically change the way we play, work, share information, and communicate (Custovic & Spahic, 2023). From the early acquisition of the *Oculus* VR system by Facebook in 2014, there has been rapid development of a variety of VR systems. As such, several research labs have addressed the uses and effects of VR technologies during the last two decades, providing evidence that many of the cognitive and emotional mechanisms governing real life also apply to experiences in VR and vice versa (Bailenson, 2018). VR has, importantly, already sustainably changed the way we conduct experimental research in many scientific domains, including social psychology, cognitive science, and neuroscience (Armougum et al., 2019; Calabro & Naro, 2019; Großekathöfer et al., 2022; Hu & Roberts, 2020; Marcum et al., 2018; Parsons et al., 2017; Yang et al., 2024; Yaremych & Persky, 2019). The many applications have highlighted the enormous potential of VR as a research tool combining high levels of experimental control with situational realism and ecological validity (see Bente, 2019; Blascovich et al., 2002; Parsons, 2015). It is surprising that researchers in the field of media psychology have widely ignored the methodological opportunities of VR, focusing almost exclusively on the socio-emotional experiences offered by the new medium and its psychological effects. Much of the existing literature on VR in media psychology explores social cognition and decision-making (Armougum et al., 2019; Yang et al., 2024; Yaremych & Persky, 2019), particularly in terms of how these processes contribute to positive outcomes within VR experiences. However, comparatively little attention has been paid to the methodological potential of VR as a research tool. The current study aims to close this void by introducing a customizable virtual research ecosystem for the study of screen media effects.

This open-access, interactive VR environment encompasses a virtual living room with a wall-mounted 65″ virtual TV on which any digital video content can be streamed. Importantly, the VR presentation software enables the seamless integration of process measures, including subjective continuous audience ratings and objective physiological response data. Being customizable, mobile, and independent of the research site, the current research platform has important advantages over “physical” lab settings. It promotes replicability by standardizing the reception context across varying research sites. Moreover, audiences’ experiences can be shielded against undesirable environmental influences and distractions (e.g., differences in room layout, lighting conditions, background noise, variability in presentation devices such as screen size or speaker quality, and inconsistencies in stimulus timing), or even systematically exposed to context factors if needed (e.g., introducing controlled interruptions or secondary response tasks). Last but not least, the entire lab can be transported in a small suitcase to any location and audience group of interest (e.g., rural and minoritized communities, individuals with disabilities, among others).

Despite these apparent advantages, a broader deployment of VR methods in media research depends on proof of concept. To demonstrate practicability and validity of the novel approach, we conducted an evaluation study comparing audience responses to emotional movie clips presented in either a physical living room (TV condition) or a virtual environment (VR condition). Specifically, we aimed to illustrate the efficiency of the VR environment in inducing feelings of spatial presence (RQ1) and, simultaneously, excluding potential side effects of prolonged VR device use in terms of virtual sickness (RQ2).

Traditional screen media, such as movies and television shows, remain a dominant format for narrative consumption and emotional engagement in everyday life. It is also one of the most thoroughly researched mediums in media psychology, thus offering a well-established baseline for evaluating emotional and physiological responses to entertainment media. By comparing traditional media-watching experiences to those in a virtual environment, the current study assesses how immersive media technology aligns with or diverges from the known patterns of media effects. The major objective of the present method evaluation study was to demonstrate that both subjective and physiological audience responses to emotional movie clips correlate significantly with audience responses assessed in comparable physical settings, and that specific response patterns expected for different emotional stimulus qualities are consistent across both reception conditions. To ensure a thorough comparison, we employed a set of movie clips categorized as scary, funny, and sad, each crafted to evoke intense emotional responses. Previous research indicates that stimuli conveying fear, joy, or sadness elicit not only varied subjective feelings but also distinct physiological responses (see Neil, 2019; Arias et al., 2020; Chang et al., 2013). For instance, in the analysis of emotionally charged televised campaigns, Lee and Lang (2009) discovered a correlation between fear and high arousal, joy and moderate arousal, and sadness and low arousal. To assess subjective and objective dimensions of the audience responses, we included continuous ratings of felt emotional intensity and psychophysiological arousal measures. We hypothesized that the two experimental settings (TV vs. VR) would demonstrate convergent validity in all process measures, as apparent in significant correlations throughout the timeline (H1), as well as consistently distinct responses to the movie clips with different emotional tones (H2). Indeed, the study examined how viewing media in a virtual environment may impact the internal versus ecological validity of audience responses.

## Method

### The VR ecosystem

The VR ecosystem is coded in Python using the VR programming platform Vizard 7.0 (Worldviz). The platform can be adapted to interface with most current VR devices. This study used Oculus Quest 2 headsets connected via Wi-Fi tethering. The 3D living room model was downloaded from Sketchfab (sketchfab.com) and slightly modified to eliminate distracting details, such as a fireplace. A virtual TV set was added to the scene on which video content was streamed as a texture. Further changes regarding details in the virtual environment aimed to replicate the real-world TV living room setting as closely as possible.

**Fig. 1:**
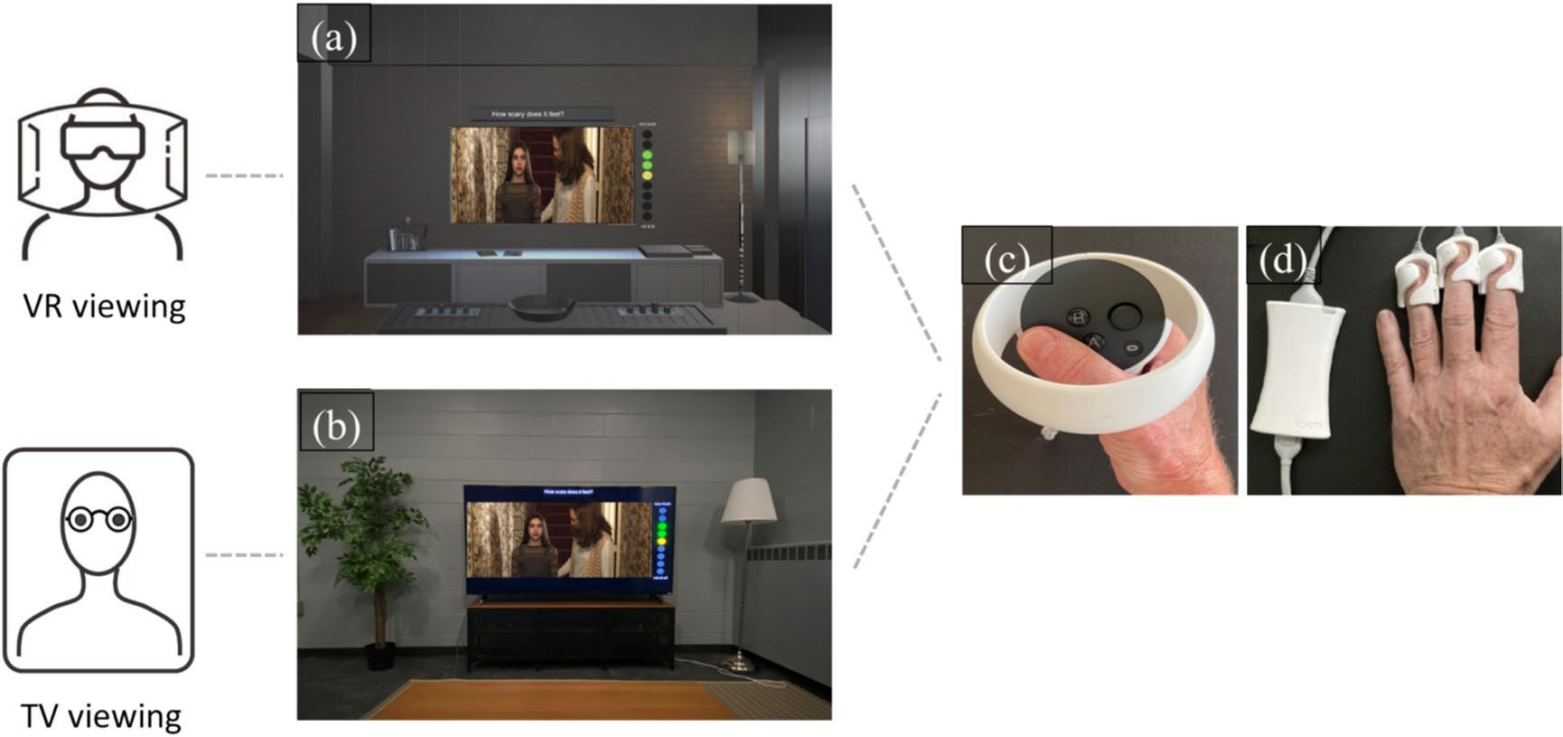
Experimental setup. (**a**) screenshot of the VR viewing condition, (**b**) image of the TV viewing condition, (**c**) Meta Quest 2 right-hand controller, (**d**) IOM1 physiological sensors

The program for the real-world TV condition used the same code, but instead of streaming the video content to the VR TV set displayed in the headset, it streamed the video to a physical 65″ screen. The software also includes an interactive nine-point gauge for continuous response measures displayed in the field of view and assessed via the Quest 2 controller or computer mouse. See complete experimental setup in Fig. 1.

### Participants

Seventy participants were recruited from a large Midwestern university student population through the college’s SONA subject pool that provided course credit for participation. The sample was predominantly White (75.7%) women (48.6%) with an average age of 20.64 years (*SD* = 4.51). See Table 1 in the supplement for more demographic information. The data for 18 participants were dropped prior to analysis due to missing or unusable physiological data capture. Importantly, these exclusions were based solely on procedural error and not participant responses. Participants were randomly assigned to both experimental conditions (*n* = 35 in VR condition, *n* = 35 in TV condition). All participants provided informed consent prior to participation.

### Stimuli

The stimulus material consisted of nine movie clips, with three clips portraying each of the three emotional tones: scary, funny, and sad. These scenes were selected with the intention to elicit intense and distinct emotional responses of thrill, amusement, and sadness. While being aware of the risk of serial effects, we kept the same presentation order for all participants in order to maximize the emotional effects and to minimize potential carryover effects between frequently iterating emotional stimulus qualities. The experiment began with the three putatively most arousing and negatively valenced scary clips, then moved to the three less arousing and positively valenced funny clips to mitigate any lingering negative arousal, and concluded with the three putatively low-arousal and negatively valenced sad clips, thereby expecting to lead to an overall gradual decrease in the audience’s arousal level. The movie clips were selected by the four authors on the basis of their face validity in conveying the three intended emotional genres (scary, funny, sad). The genres of the media stimuli were validated by IMDb.com and also carefully evaluated to ensure the selected clips were emotionally evocative, narratively coherent when clipped, and roughly comparable in length and pacing across genres. Table 1 includes a list of the selected movie titles with brief descriptions of the scenes used for the current study.

**Table 1:** Stimulus materials presented in the same order as stimuli exposure

Movie Title	Tone	Description of Scene
*The Conjuring 2* (2016)	Scary	A paranormal investigator finds a dark presence in her home.
*The Ring* (2002)	Scary	Against his friend’s discretion, a man watches a cursed videotape.
*Guest* (2020)	Scary	A disturbed woman harms herself when she encounters a strange creature.
*Hot Rod* (2007)	Funny	An aspiring stunt man is frustrated and takes to the forest to let off steam.
*The Other Guys* (2010)	Funny	Two detectives arrive at an old flame’s house to gather information for their case.
*I Think You Should Leave with Tim Robinson* (2019)	Funny	A car crashes into a retail store, and the bystanders attempt to find the culprit.
*My Girl* (1991)	Sad	A grieving child tries to attend her best friend’s funeral before talking to her teacher.
*Marley & Me* (2008)	Sad	A father says goodbye to his senior dog, and the family reflects on fond memories.
*The Green Mile* (1999)	Sad	A White death row supervisor executes a wrongfully convicted Black man.

*Note. I Think You Should Leave with Tim Robinson* is a sketch comedy television series and was still included as a funny stimulus as the scene length was three minutes, the show is tagged as a comedy on IMDb, and, importantly, it is very funny

Three clips portraying each emotion were presented in blocks, and the blocks were always shown in the same order (i.e., scary – funny – sad) to allow frame-by-frame averaging of the individual time series. To obtain baseline measures and to reset arousal levels after each presentation block, a two-minute relaxation video was presented at the start of the experiment, between blocks, and at the end of stimulus exposure. The total length of stimulus material was approximately 36 minutes. It is important to note that all the stimulus clips were downloaded locally to the laptop, and the laptop and VR headset were connected wirelessly through a router that did not require internet access. There were no inconsistencies in rendering times of the digital video; the stimulus playing times were identical, as we could see from stamped records. Thus, the only limit to running this program and the stimuli associated with it is having the base processing power to create a simple VR environment (which almost all laptops with dedicated graphics can do in this era).

### Process measures

Continuous response measures during stimulus reception included real-time ratings (RTR) of experienced emotional intensity and physiological data. Continuous audience ratings of emotional intensity were acquired via the right-hand Meta Quest 2 controller (VR condition) or a wireless computer mouse (TV condition). Depending on which clip was shown, participants were prompted to answer the question “How scary does it feel?” or “How funny does it feel?” or “How sad does it feel?” on a nine-point scale (from 1 = *not at all* to 9 = *very much*) displayed on the right side of the TV screen. Images of the TV screens in both experimental settings are shown in Fig. 1a and b.

Physiological data included skin conductance level (SCL) and the photoplethysmographic (PPG) measure of peripheral blood flow from which inter-beat interval (IBI, equivalent to heart rate) and pulse volume amplitude (PVA) were derived (see the “Pre-processing of audience responses” subsection). PVA indicates vasoconstriction and vasodilation of the peripheral vessels (in our case, the fingertips) reflecting tension and relief in the autonomic nervous system. PVA has recently been successfully used to identify audience responses in particular to suspenseful movie content (Baldwin & Bente, 2021; Bente et al., 2022).

Physiological data were acquired via finger-clip sensors (*iom1*, Lightstone, see Fig. 1d), attached to the nondominant hand. Data were recorded at 30 Hz through a C +  + program based on the *iom1* SDK (software development kit). The main stimulus presentation program (Python) started and stopped the physio recording and sent the continuous rating data to the physio recording program to be stored in sync with the physio data at 30 Hz.

### Survey measures

The pre-experimental survey consisted of sociodemographic items and established scales to evaluate participants’ affect, cognition, personality, and immersive tendencies (not reported here). The post-experimental survey consisted of two parts: (1) a stimulus check for all participants and (2) a set of scales for participants in the VR condition to examine their experience wearing the Quest headset. First, participants were asked to rate the intensity of the conveyed emotion (scary, funny, sad) on a scale from 0 to 100 using an on-screen slider and to indicate whether they had seen the movie before. VR participants then completed the post-experimental survey. See Table 2 in the supplement for survey measure details. All surveys were administered on a PC through Qualtrics.

### Procedure

Upon arrival, participants completed a pre-experimental Qualtrics survey, assessing participants’ demographics, personality, as well as media usage and engagement habits. After being assigned to one of the experimental conditions, participants were seated in a living room-style lab on a comfortable couch. Finger sensors for physiological assessment were then applied to the nondominant hand (see Fig. 1d). Participants in the VR condition were then instructed on how to use the A and B buttons of the Quest controller, while participants in the TV condition were taught to use the left and right mouse clicks to scale up and down the felt emotional intensity (see Fig. 1c). Participants in the VR condition then put on and adjusted the VR headset, in which they could see a virtual replica of the physical space, while participants in the TV condition had an open view to the 65″ TV set. Next, the nine video clips were presented always in the same order (scary, funny, and sad clips with intermittent relaxation phases) to allow seamless averaging of the individual response time series. After stimulus presentation, participants completed the Qualtrics post-experimental survey.

### Pre-processing of audience responses

RTR, SCL, and PPG were pre-processed using a custom python program. Using the HeartPy library (https://github.com/paulvangentcom/heartrate_analysis_python/), the program performed peak detection in the PPG raw data. Using the peak time stamps provided by HeartPy, time series for the IBI and PVA were calculated for the different segments of the stimulus. Together with the corresponding segments of RTR and SCL, the resulting time series were concatenated, ensuring the identical data sample length for each participant and each stimulus segment. Dropping the first 10 s of recordings (to account for filter stabilization and orienting responses), baseline correction was applied to IBI, PVA, and SCL data. Data for PVA and SCL were further de-trended to exclude common level drifts that can cause an inflation of correlations. All physio data were then *z*-transformed and submitted to a second-order lowpass filter with a 0.5 cutoff frequency. Averages of the different segments were subsequently used for statistical analyses.

## Results

### Stimulus check

**Fig. 2:**
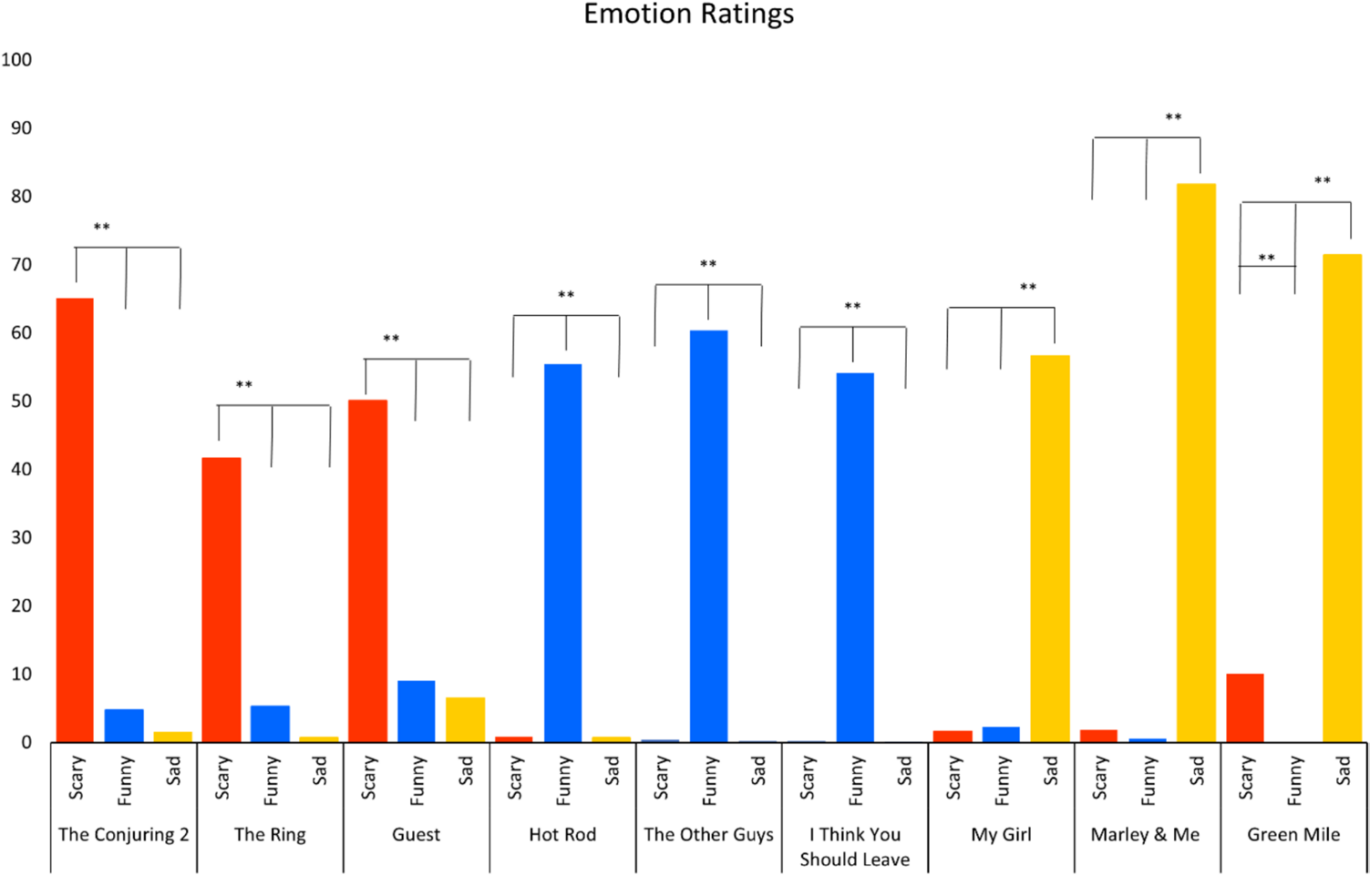
Emotion ratings for all nine movie clips across the three emotions. *Note.* ** p-value < 0.001

To test the typicality of each clip within the intended emotion category, nine separate repeated measures analyses of variance (ANOVAs) were conducted for the three emotion ratings provided on the 0–100-point scale (scary, funny, sad). Results showed that all clips were rated significantly higher on the target emotion than on either of the other emotions (see Fig. 2; ANOVA results can be found in Tables 3 and 4 in the supplement). Mixed feelings were only found in a few cases with low intensity ratings for the nontarget emotions. Specifically, the sad movie, *The Green Mile*, revealed feelings of scariness, and all scary movies, *The Conjuring 2*, *The Ring*, and *Guest*, were perceived as slightly funny.

### User experience in the VR condition

**Fig. 3:**
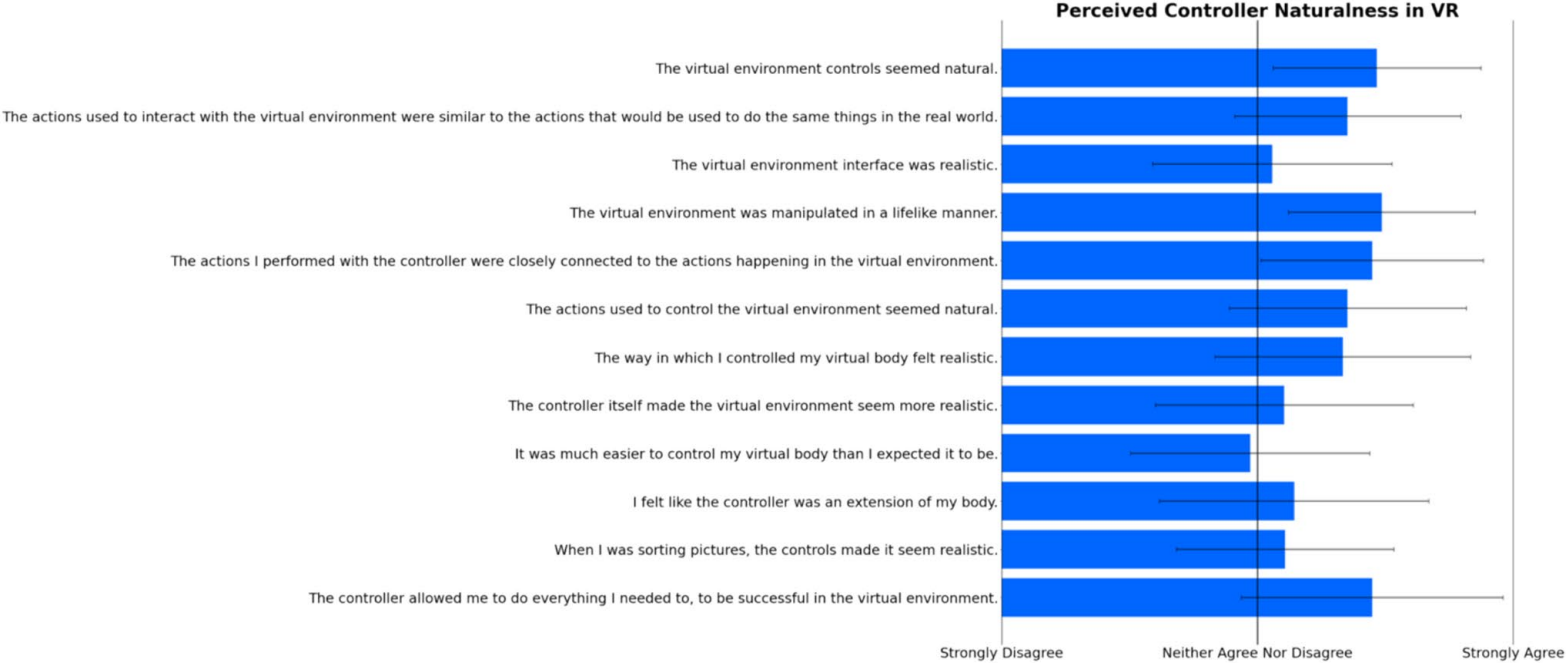
Response averages for Perceived Controller Naturalness items

**Fig. 4:**
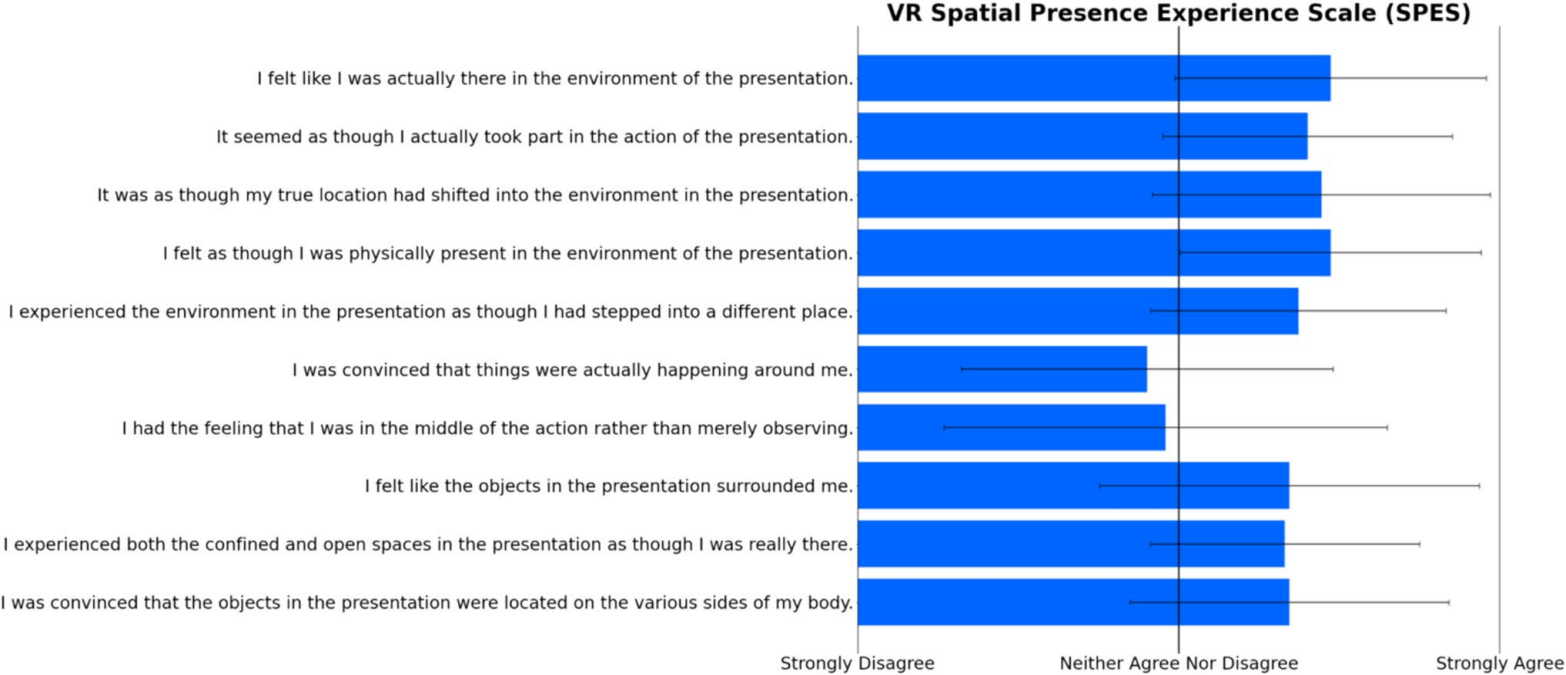
Response averages for Spatial Presence Experience Scale items

**Fig. 5:**
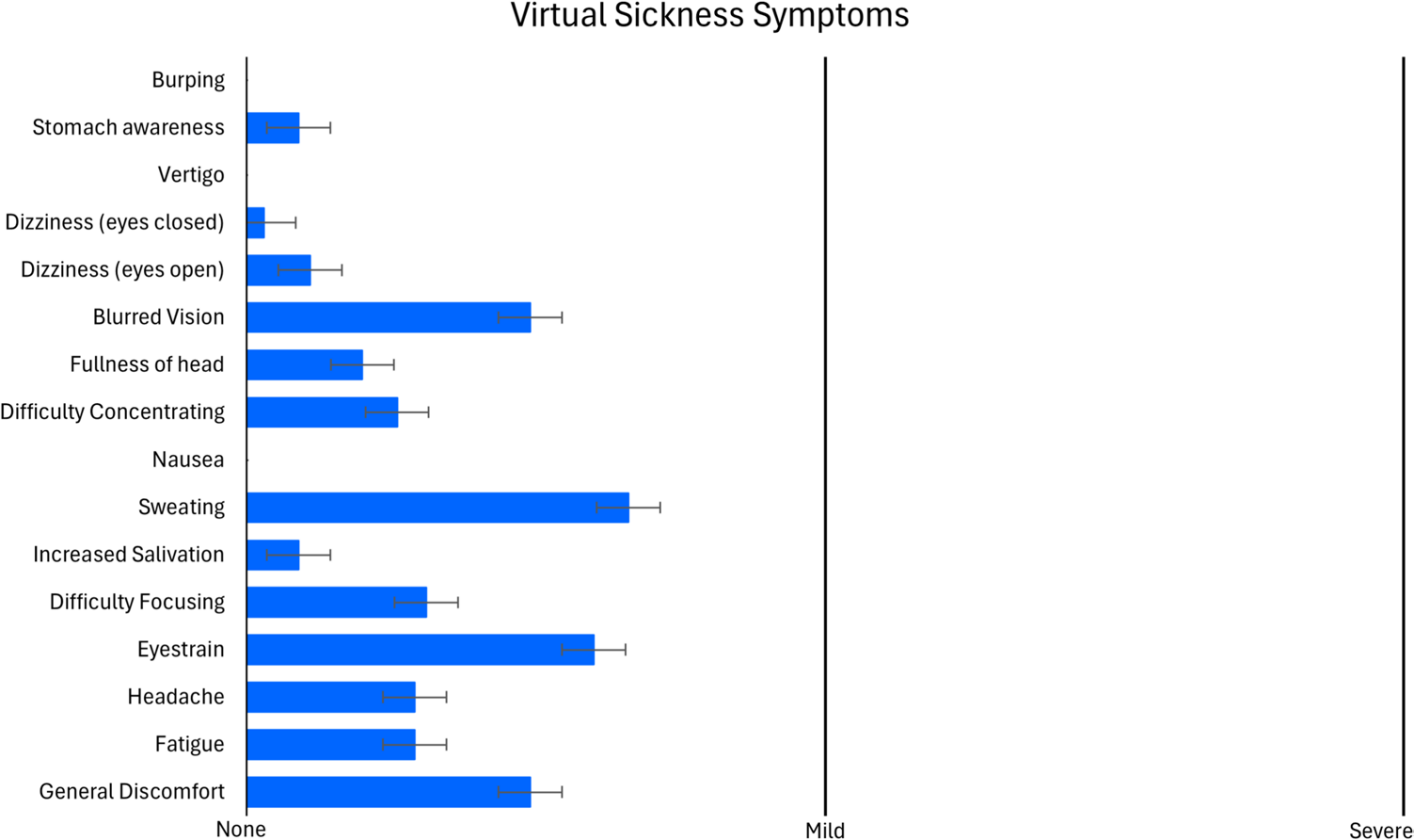
Response averages for VR Sickness Questionnaire items. *Note.* The response options were reduced from the original four-point scale (none, mild, moderate, severe) to a three-point scale (none, mild, severe)

After the virtual TV viewing session, participants provided responses about their user experience in the VR headset. A one-sample *t*-test was conducted for perceived controller naturalness (McGloin et al., 2011), testing the average across all items against the scale mean (4 = *neither agree nor disagree*). Results showed that the values deviated significantly from scale mean (*M* = 4.83, *SD* = 0.77; *t*(34) = 6.38,* p* < 0.001), indicating that the controller felt intuitive and natural for participants to use for RTR in the virtual environment. See Fig. 3 for a display of the average responses per item. Another one-sample *t*-test was conducted for the Spatial Presence Experience Scale (Hartmann et al., 2016), testing the average across all items against the scale mean (3 = *neither agree nor disagree*). Results showed that the values deviated significantly from scale mean (*M* = 3.60, *SD* = 0.73; *t*(34) = 4.84,* p* < 0.001), indicating high levels of perceived spatial presence in the virtual room, which further provides the authentic viewing experience as well as the real space. Figure 4 displays the average responses per item. We also conducted a descriptive analysis of the Simulator Sickness Questionnaire (Kennedy et al., 1993). Figure 5 displays the average scores for each item, indicating that participants did not experience any notable feelings of discomfort or sickness symptoms during the more than half-hour session of VR exposure in a highly controlled and undisturbed environment. For a clearer picture of the response patterns and central tendency per item, see raw count distributions of each item for perceived controller naturalness, spatial presence, and virtual sickness in the supplement (Fig. 1, 2, 3, Tables 5 and 6).

### Audience responses across stimuli and conditions

**Fig. 6:**
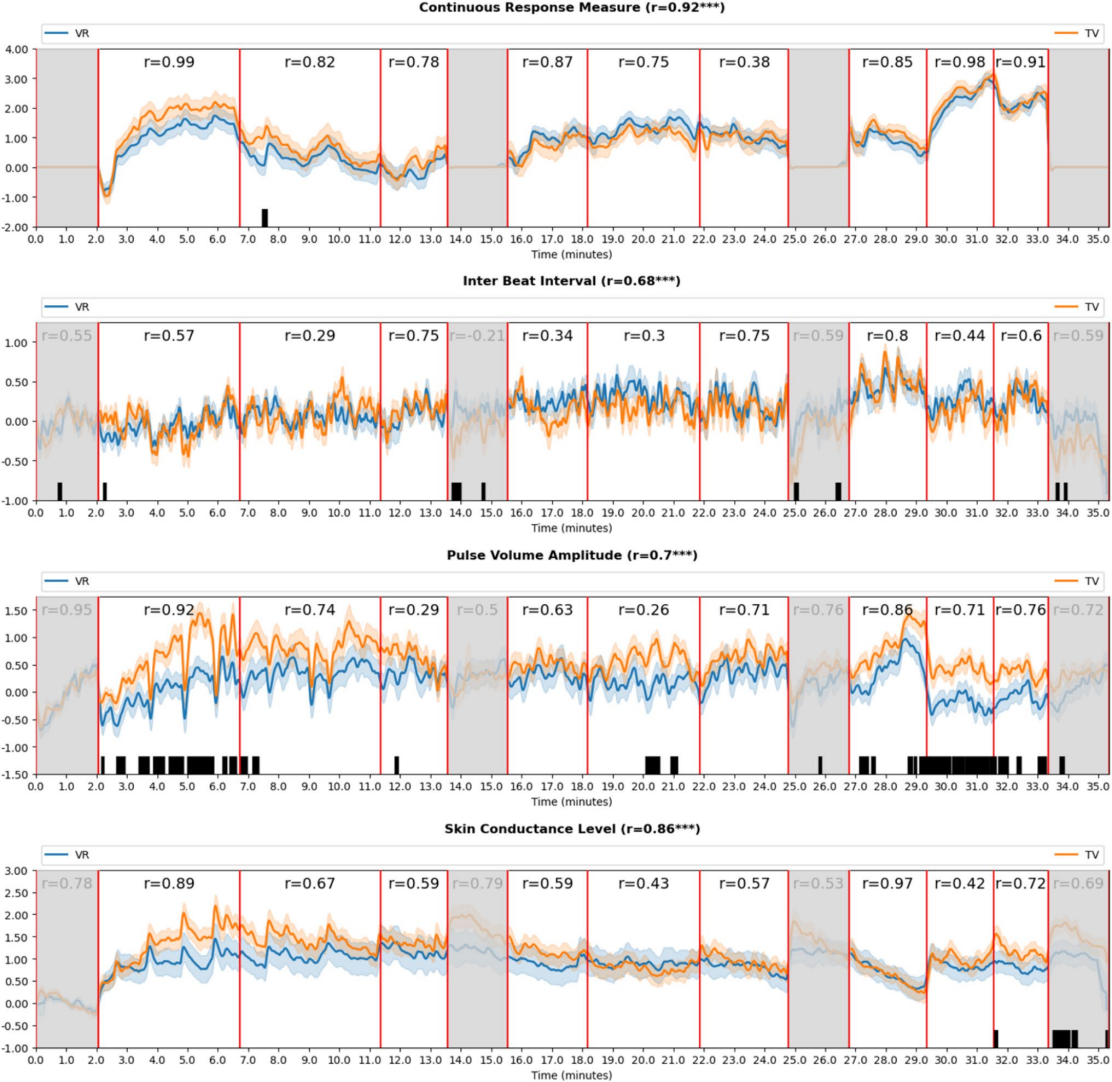
Averaged response time series for the two experimental groups, including overall and clip-wise correlations as well as frame by frame T-test comparisons. *Note. *The time windows align with the duration of each individual movie clip in the stimulus sequence. That is, each window represents the full runtime of one of the nine movie clips with the four gray windows representing relaxation phases

Figure 6 displays the time course of the averaged audience responses in both groups for the four parameters CRM, IBI, PVA, and SCL. The time graphs follow the order of the stimulus exposure (see Table 1 for stimulus order). The gray boxes represent the relaxation phases. The figure also indicates the Pearson correlations between the time series of both groups for the whole session (see graph titles) as well as for the nine clips (see graph area). The bar codes at the bottom of each of the four graphs indicate a significant difference (*p* < 0.05) between the groups at the respective point in time. The time graphs and the correlations both demonstrate well-aligned response patterns between the VR and TV condition, with highly significant overall correlations (*p* < 0.001) in all parameters (CRM: *r* = 0.92; IBI: *r* = 0.68; PVA: *r* = 0.7; SCL: *r* = 0.86), thus supporting H1. Correlations on the clip level were all significant given the large sample size but also showed considerable variation in the explained variance. Specifically, IBI and PVA show lower correlations during one of the scary and one of the funny clips. An explanation is provided in the “Discussion” section.

Subsequent *t*-test comparisons for each time point revealed differences for whole clips in PVA for the first scary clip, *The Conjuring 2*, and the second sad clip, *Marley & Me*. Further differences in PVA were found for the beginning of the second scary clip, *The Ring*, as well as parts of the first sad clip, *My Girl*, and parts of the third sad clip, *The Green Mile*. 

To differentiate between the influence of the emotion category and the experimental condition, we further conducted repeated measures ANOVAs for the four parameters with condition as between-subjects factor and emotional tone as within-subjects factor, using the average scores across the three clips per category (see Table 2). We found no main effect of condition on any of the measures, but a main effect of emotion for CRM (*F*(2, 67) = 28.23, *p* < 0.001), IBI (*F*(2, 67) = 8.59, *p* < 0.001), and SCL (*F*(2, 67) = 13.42, *p* < 0.001). Only a marginally significant main effect of emotional tone was found for PVA (*F*(2, 67) = 2.68, *p* < 0.07). ANOVA results overall confirm H1 and H2.

**Table 2:** Repeated measures ANOVAs for the process measures with stimulus category (emotion) as within-subjects factor and viewing condition (VR vs. TV) as between-subjects factor

		*SS*	*df*	*MS*	*F*	*p*
CRM	Emotion	57.15	2	28.57	28.23	<.001
	Emotion * condition	2.19	2	1.09	1.08	.34
	Condition	.67	1	.67	.24	.62
IBI	Emotion	2.50	2	1.25	8.59	<.001
	Emotion * condition	.13	2	.06	.46	.63
	Condition	.09	1	.09	.10	.75
PVA	Emotion	.85	2	.42	2.68	.07
	Emotion * condition	.40	2	.20	1.25	.28
	Condition	8.76	1	8.76	3.59	.06
SCL	Emotion	5.50	2	2.75	13.42	<.001
	Emotion * condition	.40	2	.20	.98	.376
	Condition	1.14	1	1.14	.20	.65

## Discussion

This research demonstrates the utility and validity of a VR ecosystem for the study of emotional screen media effects. The current study compared a combination of subjective and objective physiological effects of film clips with distinct emotional tones (scary, funny, sad) shown in either a physical living room environment (TV condition) or in a virtual environment (VR condition). Three clips of each emotional tone (3 × 3) were presented consecutively with short relaxation pauses while continuously measuring RTR, IBI, PVA, and SCL.

In terms of general effectiveness and acceptability of the experimental VR setting, data revealed that participants in the VR condition felt they viewed videos in a real, natural, and life-like TV viewing environment. They also reported significant levels of spatial presence in the virtual living room and did not experience any noteworthy virtual sickness symptoms. This is a promising result regarding the further use of the ecosystem by indicating its effectiveness in creating an ecologically valid reception setting and, importantly, demonstrating that even longer stimulus exposures via headsets (in our case, more than half an hour) are not putting any psychological or physical strain on the participants. It is important to note that our participants were comfortably seated and did not move in the space. While this is a typical TV or film reception situation, other applications with free movement in space would need to be tested again for cybersickness effects.

A treatment check revealed that the clips were perceived as typical representatives of the respective emotional category. As expected, we found significant correlations between the conditions for all four parameters across the whole session as well as at the clip level. Convergent validity of both viewing formats was confirmed by the fact that response differences between the stimulus types were consistent across participants in both conditions.

Additionally, the PVA parameter showed significant variations on the scene level. As shown in recent studies, peaks in vasoconstriction occur when uncertainty and anticipatory suspense are built up, and a subsequent drop—indicating vasodilation—can be observed when tension is relieved through a specific resolution (see Bente et al., 2022). This effect was particularly pronounced during the scary movie, *The Conjuring 2*, which used lighting and music to induce uncertainty and the anticipation of most scary events. Importantly, this effect could be observed in both experimental conditions, again supporting the use of the VR ecosystem in further media effect studies.

## Limitations

All participants viewed the movie clips in a fixed order: scary, funny, sad. This order was chosen for intentional emotional sequencing, although we understand this may introduce a possible order effect. By embedding the clips within the context of the same genre, we reduced the likelihood of carryover effects when moving from one genre to the next. That is, audience responses to the second or third clips within each genre category should be clean and uninfluenced by their previous media exposure. Moreover, our primary analyses focused on audience responses within each emotional genre rather than on direct comparisons across genres. This study prioritized testing (1) similar patterns in process measures between the VR and TV conditions and (2) distinct audience responses to the three types of emotionally varying movie clips, upon which the fixed order of the movie clips was unlikely to systematically bias our main findings.

Although participants reported that their VR experience felt very realistic and that it unfolded in a manner they perceived as natural, VR experiences are not yet completely equivalent to real-life TV viewing experiences. Instead, VR serves as a highly simulated representation of real-life scenarios. This could be a double-edged sword for future research. On the one hand, the highly controlled VR environment ensures that observed effects can be more confidently attributed to the experimental manipulations, while the ecological validity can also be considered since the idea of the VR environment comes from real-world TV viewing scenario. That is, The VR-TV setting provides a solid way for researchers to balance both internal validity and ecological validity. However, on the other hand, future research should consider participants' expectation and familiarity with this emerging medium of virtual reality, as well as how individual experience affects their behavioral responses (e.g., participants may experience visual distraction due to curiosity about the environment they have never experienced before). For instance, this study’s sample comprised college students, a population that may be more technologically savvy and comfortable navigating a virtual space compared to the general population. This limitation may have contributed to the generally positive evaluations of the VR experience. Nevertheless, the results of this study still demonstrate the convergent validity of VR and real-life environments, which can be broadly applied to examining responses to screen media.

Notably, frame-by-frame *t*-test comparisons revealed a few phases of significant level differences in the PVA parameter between experimental conditions. The first scary clip (*The Conjuring 2*) and the second sad clip (*Marley & Me*) showed higher levels of vasoconstriction (lower PVA level), indicating higher levels of body tension. Interestingly, these two clips were also rated highest in perceived emotional intensity within their respective emotion categories. This underpins the utility of the PVA parameter in indicating the intensity of affective responses (see Baldwin & Bente, 2021), suggesting that in cases of extreme emotional responses, the VR condition can lead to more physiological tension than the real-world setting. One could speculate that, in those cases, audiences usually mitigate too strong feelings by redirecting their attention to the physical reality surrounding them. However, this is not quite possible in VR, where the boundaries between the TV media content and the environment are blurred. Consistent with this interpretation, continuous level differences in PVA were also found for the sad clip, *Marley & Me*, which was probably the most tear-provoking one of the three clips shown. These insights tie back to a larger discussion regarding the implications of the study’s convergent validity. The media processing and effects that take place in a virtual environment may not always generalize to what happens when people consume traditional media. A virtual environment is more controlled than a physical lab, so we may expect stronger but less natural effect sizes when testing the effects of certain screen media.

## Implications for further research

The current research demonstrates that VR can serve as a reliable tool for studying media effects given its confirmed usability and convergent validity of VR in eliciting emotional responses comparable to those in real-world settings. However, some findings, such as the PVA parameter differences between conditions, open doors to exploring how various environmental factors influence emotional engagement with media content. Moving forward, additional research should further explore the nuanced differences in emotional responses between traditional and VR environments. Exploring factors like immersion, need for cognition and affect, and prior experience or familiarity with VR may illuminate how these variables interact for one’s media experience. Indeed, these variables were measured in the current study but will be analyzed and reported in a separate paper. Furthermore, in studying the use of VR in screen media research, individual differences, particularly prior VR experience, may influence comfort and emotional engagement in the virtual environment and should be tested beyond college students enrolled in a communications course.

The key advantage of employing a VR lab environment lies in the ability to tightly control the core elements of an experimental manipulation across sites. Traditionally, aspects of the participants’ physical surroundings may vary slightly, such as room lighting, background noise, or experimenter presence. However, these can typically be minimized or held constant through detailed protocols that ensure quiet lab environments and controlled instructions. It is important to standardize aspects within a VR ecosystem to scientifically explore how residual elements of the environment may interact with media experiences. Future studies can leverage VR’s capability for experimental control by manipulating environmental cues systematically, which can further elucidate the causal mechanisms underlying media effects. Moreover, the portability and standardization advantages of a VR ecosystem present new possibilities for larger studies across diverse populations and contexts. That is, this scalability could facilitate cross-cultural studies or investigations into how different demographic groups respond to media content, thus providing insights into universal versus culturally specific emotional reactions.

## Conclusion

Overall, our results demonstrate convergent validity between a traditional TV setting and a VR-based paradigm and, thus, support the use of our VR ecosystem in experimental media research. The advantages of the platform go far beyond the mentioned combination of experimental control and ecological validity. VR affords the standardization of experimental settings when using different study sites and bringing the lab to people at remote locations or with reduced mobility. The present platform is customizable and allows seamless integration of experimental variations into the virtual environment. For instance, secondary response tasks (SRTs) could be part of the setting, thus overcoming a major criticism that the SRT is presented on the same screen as the stimuli. Importantly, VR headsets can accommodate various objective measures, such as eye tracking, electroencephalography (EEG), physiological sensors, and lower face movements, as well as gross movement of the head and the body. These measures are of significant value for media effect studies, and the demonstration of convergent validity here can spawn a strong motivation to intensely use this or comparable VR ecosystems.

## Supplementary Information

Below is the link to the electronic supplementary material.

Supplementary file1 (DOCX 207 KB)

## Data Availability

Open Data and Materials: A detailed output of all the process data results as well as the Python program for analysis can be found at https://github.com/CARISMA-Lab/Watching-Movies-in-VR.git.

## Code availability

Open Code: The information needed to reproduce our virtual living room environment is available at https://github.com/CARISMA-Lab/Watching-Movies-in-VR.git.

## Declarations

### Open practices statement

This study was not preregistered. In accordance with TOP Level 2 guidelines, all data, materials, and code can be accessed in our trusted GitHub repository at https://github.com/CARISMA-Lab/Watching-Movies-in-VR.git. Please contact the corresponding author, Faith A. Delle (dellefai@msu.edu), for further inquiries.
