# Supplementary material for: Watching movies in VR: A research ecosystem for the study of screen media effects
Source: Behav Res Methods. 2025 Jul 25;57(9):238. doi: 10.3758/s13428-025-02750-y (PMC12296782; doi:10.3758/s13428-025-02750-y)

SUPPLEMENTARY INFORMATION

Table 1.

*Demographic information.*

|  | **VR Condition** | | **TV Condition** | |
| --- | --- | --- | --- | --- |
|  | Count | Percentage | Count | Percentage |
| *Gender* |  |  |  |  |
| Agender | 0 | 0% | 3 | 4.3% |
| Man | 14 | 20.0% | 13 | 18.6% |
| Woman | 16 | 22.9% | 18 | 25.7% |
| Non-binary | 1 | 1.4% | 0 | 0% |
| Prefer not to answer | 4 | 5.7% | 1 | 1.4% |
| *Race/Ethnicity* |  |  |  |  |
| White | 28 | 40% | 25 | 35.7% |
| Black | 4 | 5.7% | 5 | 7.1% |
| Asian | 3 | 4.3% | 9 | 12.3% |
| Middle Eastern | 1 | 1.4% | 0 | 0% |
| Mixed race | 0 | 0% | 1 | 1.4% |
| Latinx | 1 | 1.4% | 1 | 1.4% |

*Note*. Percentages are based on the number of participants selecting the responses and may total more than 100%.

Table 2.

*Survey measures.*

| **Scale Name** | **Citation** | **Example Item** | **# of Items** | **Response Type** | **M** | **SD** | **Α** |
| --- | --- | --- | --- | --- | --- | --- | --- |
| *Pretest Measures* | | | | | | | |
| Need for Affect | Appel et al. (2012) | “I feel that I need to experience strong emotions regularly.” | 10 | 1 = *strongly disagree* to 7 = *strongly agree* | 4.97 | .93 | .85 |
|  |  |  |  |  |  |  |  |
| Need for Cognition | Lins de Holanda Coelho et al. (2020) | “I would prefer complex to simple problems.” | 6 | 1 = *strongly disagree* to 7 = *strongly agree* | 4.64 | .95 | .75 |
|  |  |  |  |  |  |  |  |
| Big Five (short version) | Soto & John (2017) | “I am someone who… worries a lot.” | 30 | 1 = *strongly disagree* to 5 = *strongly agree* |  |  |  |
|  |  |  |  |  |  |  |  |
| Immersive Tendencies Questionnaire | Witmer & Singer (1998) | “Do you easily become deeply involved in movies or tv dramas?” | 18 | 1 = *never*, 4 = *occasionally*, and 7 = *often* | 4.75 | .75 | .76 |
| *Posttest Measures* | | | | | | | |
| Perceived Controller Naturalness | McGloin et al. (2011) | “The virtual environment was manipulated in a lifelike manner.” | 12 | 1 = *strongly disagree* to 7 = *strongly agree* | 4.81 | .77 | .80 |
|  |  |  |  |  |  |  |  |
| Spatial Presence Experience Scale | Hartmann et al. (2016) | “I felt like I was actually there in the environment of the presentation.” | 10 | 1 = *I do not agree at all* to 5 = *I fully agree* | 3.60 | .73 | .88 |
|  |  |  |  |  |  |  |  |
| Simulator Sickness Questionnaire | Kennedy et al. (1993) | fatigue, headache, eyestrain | 16 | 1 = *none*, 2 = *mild*, and 3 = *severe* | 1.24 | .20 | .75 |

*Note.* The three posttest measures – Perceived Controller Naturalness, Spatial Presence Experience Scale, and the Simulator Sickness Questionnaire – were the only scales analyzed for the current study. The remaining scales will be analyzed and reported in a separate paper.

Table 3.

*Repeated measures ANOVAs for each movie clip as within-subject factor on emotional tone ratings from stimulus check.*

|  |  | *SS* | *df* | *MS* | *F* | *p* |
| --- | --- | --- | --- | --- | --- | --- |
| Scary | *The Conjuring 2* | 179637.26 | 2 | 89818.63 | 270.49 | < .001 |
|  | *The Ring* | 70623.62 | 2 | 35311.81 | 114.28 | < .001 |
|  | *Guest* | 79260.07 | 2 | 39630.03 | 89.56 | < .001 |
| Funny | *Hot Rod* | 129256.90 | 2 | 64628.45 | 273.12 | < .001 |
|  | *The Other Guys* | 165922.01 | 2 | 82961.00 | 370.36 | < .001 |
|  | *I Think You Should Leave* | 129996.18 | 2 | 64998.09 | 287.04 | < .001 |
| Sad | *My Girl* | 137483.77 | 2 | 68741.88 | 292.24 | < .001 |
|  | *Marley & Me* | 303166.31 | 2 | 151583.15 | 835.81 | < .001 |
|  | *The Green Mile* | 205778.98 | 2 | 102889.49 | 446.87 | < .001 |

Table 4.

*Post hoc comparisons of emotion ratings on each movie clip.*

|  |  | *Mean Diff.* | *SE* | *t* | *P_holm_* |
| --- | --- | --- | --- | --- | --- |
| *The Conjuring 2* | Sad x Funny | -3.27 | 3.08 | -1.06 | 0.29 |
|  | Sad x Scary | -63.61 | 3.08 | -20.65 | < .001 |
|  | Funny x Scary | -60.34 | 3.08 | -19.59 | < .001 |
| *The Ring* | Sad x Funny | -4.57 | 2.97 | -1.53 | .12 |
|  | Sad x Scary | -40.98 | 2.97 | -13.79 | < .001 |
|  | Funny x Scary | -36.41 | 2.97 | -12.25 | < .001 |
| *Guest* | Sad x Funny | -3.97 | 3.66 | -1.084 | .28 |
|  | Sad x Scary | -44.28 | 3.66 | -12.09 | < .001 |
|  | Funny x Scary | -40.31 | 3.66 | -11.01 | < .001 |
| *Hot Rod* | Sad x Funny | -54.67 | 2.69 | -20.26 | < .001 |
|  | Sad x Scary | -.12 | 2.69 | -.04 | .96 |
|  | Funny x Scary | 54.55 | 2.69 | 20.21 | < .001 |
| *The Other Guys* | Sad x Funny | -60.17 | 2.54 | -23.61 | < .001 |
|  | Sad x Scary | -.23 | 2.54 | -.09 | .92 |
|  | Funny x Scary | 59.94 | 2.54 | 23.52 | < .001 |
| *I Think You Should Leave* | Sad x Funny | -53.97 | 2.60 | -20.75 | < .001 |
|  | Sad x Scary | -.04 | 2.60 | -.01 | .98 |
|  | Funny x Scary | 53.92 | 2.60 | 20.74 | < .001 |
| *My Girl* | Sad x Funny | 54.33 | 2.61 | 20.80 | < .001 |
|  | Sad x Scary | 55.00 | 2.61 | 21.06 | < .001 |
|  | Funny x Scary | .66 | 2.61 | .25 | .79 |
| *Marley & Me* | Sad x Funny | 81.22 | 2.27 | 35.68 | < .001 |
|  | Sad x Scary | 79.95 | 2.27 | 35.12 | < .001 |
|  | Funny x Scary | -1.27 | 2.27 | -.55 | .57 |
| *The Green Mile* | Sad x Funny | 71.37 | 2.58 | 27.63 | < .001 |
|  | Sad x Scary | 61.23 | 2.58 | 23.70 | < .001 |
|  | Funny x Scary | -10.14 | 2.58 | -3.92 | < .001 |

Figure 1.

*Frequency of responses for Perceived Controller Naturalness items.*


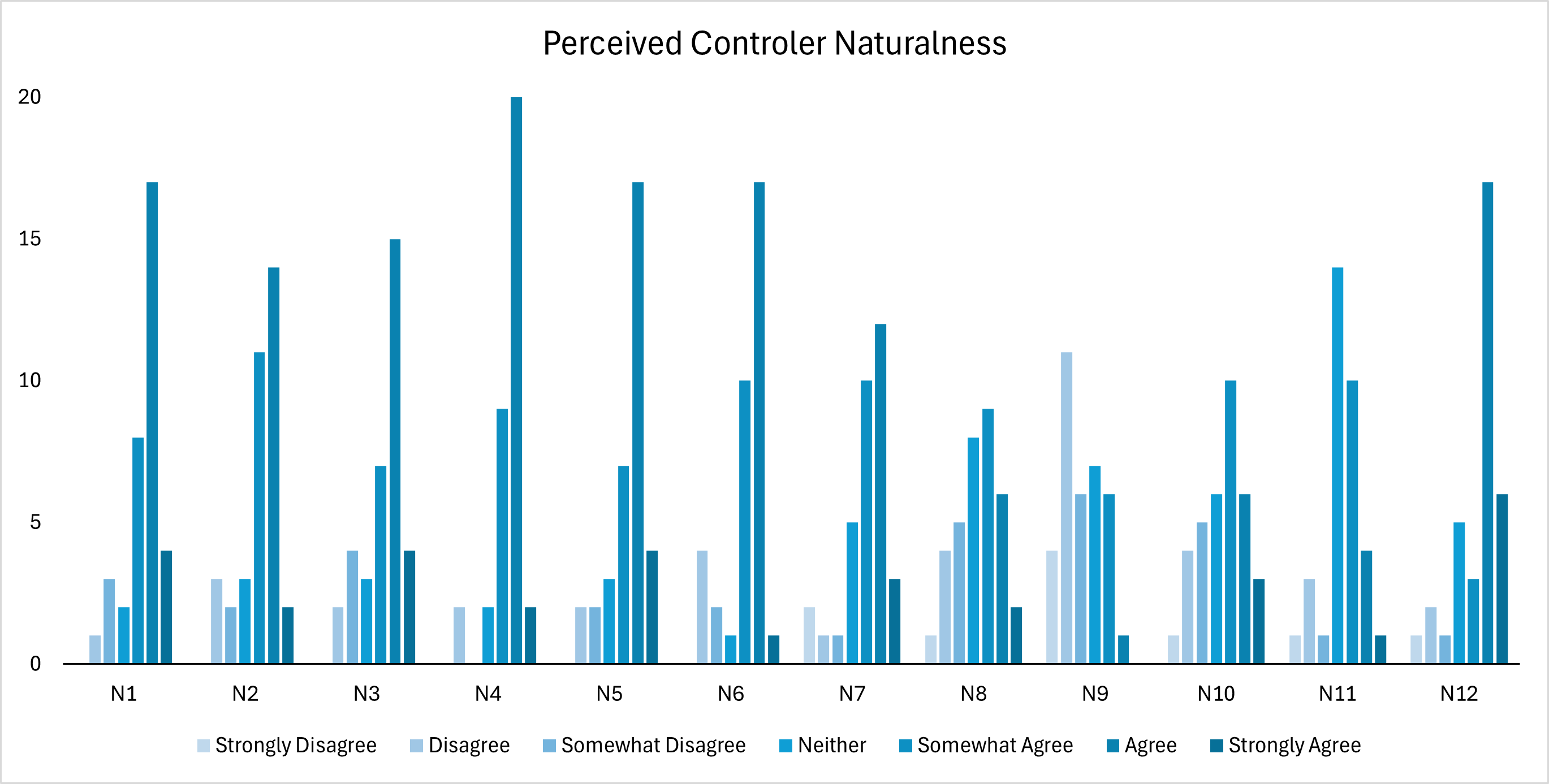


Table 5.

*Perceived Controller Naturalness items.*

| **Item** | **Description** |
| --- | --- |
| N1 | The virtual environment controls seemed natural. |
| N2 | The actions used to interact with the virtual environment were similar to the actions that would be used to do the same things in the real world. |
| N3 | The virtual environment interface was not realistic (reversed item). |
| N4 | The virtual environment was manipulated in a lifelike manner. |
| N5 | The actions I performed with the controller were closely connected to the actions happening in the virtual environment. |
| N6 | The actions used to control the virtual environment seemed natural. |
| N7 | The way in which I controlled my virtual body felt realistic. |
| N8 | The controller itself made the virtual environment seem more realistic. |
| N9 | It was much more difficult to control my virtual body than I expected it to be. |
| N10 | I felt like the controller was an extension of my body. |
| N11 | When I was sorting pictures, the controls made it seem realistic. |
| N12 | The controller allowed me to do everything I needed to, to be successful in the virtual environment. |

Figure 2.

*Frequency of responses for Spatial Presence Experience items.*


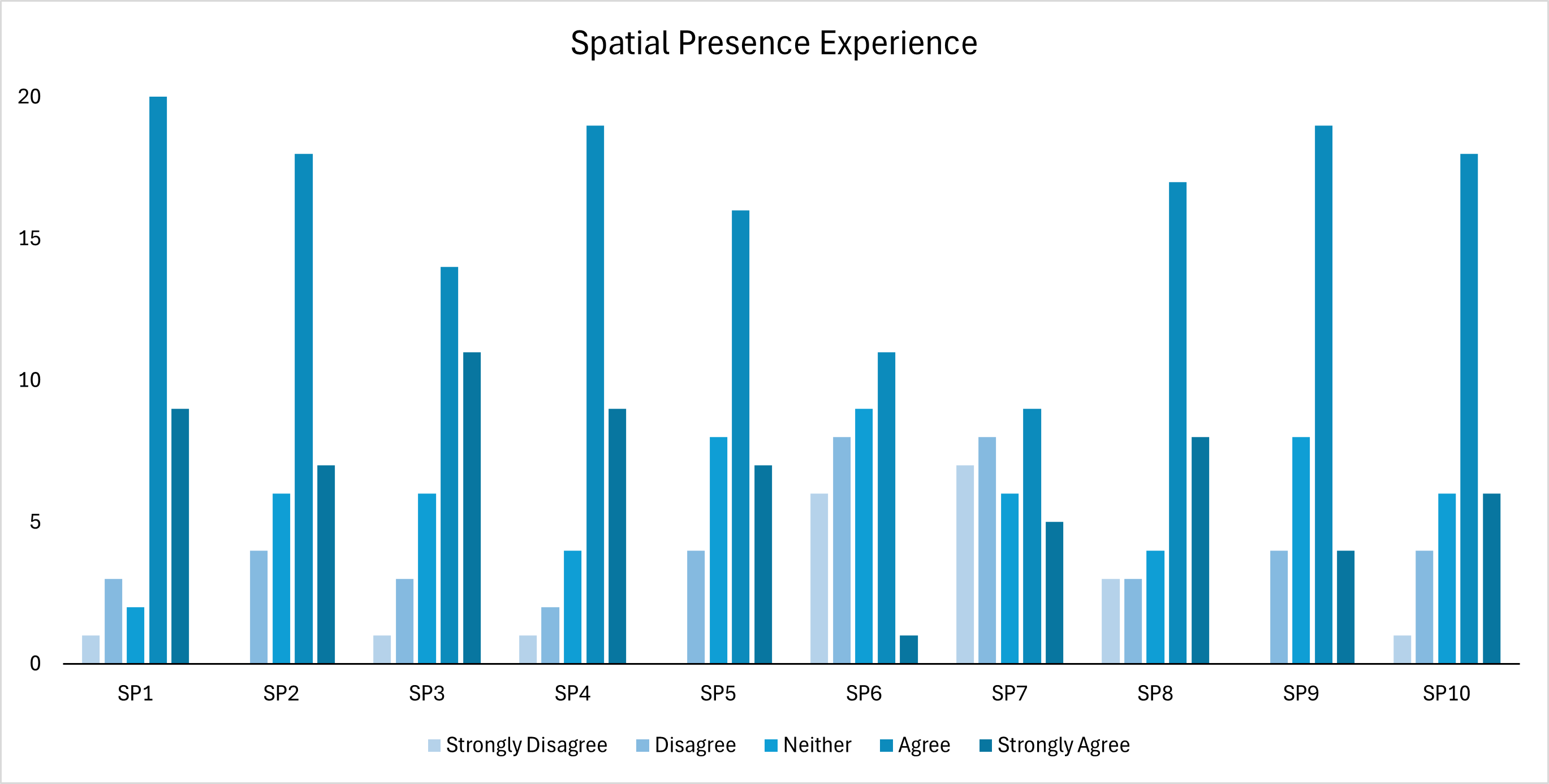


Table 6.

*Spatial Presence Experience items.*

| **Item** | **Description** |
| --- | --- |
| SP1 | I felt like I was actually there in the environment of the presentation. |
| SP2 | It seemed as though I actually took part in the action of the presentation. |
| SP3 | It was as though my true location had shifted into the environment in the presentation. |
| SP4 | I felt as though I was physically present in the environment of the presentation. |
| SP5 | I experienced the environment in the presentation as though I had stepped into a different place. |
| SP6 | I was convinced that things were actually happening around me. |
| SP7 | I had the feeling that I was in the middle of the action rather than merely observing. |
| SP8 | I felt like the objects in the presentation surrounded me. |
| SP9 | I experienced both the confined and open spaces in the presentation as though I was really there. |
| SP10 | I was convinced that the objects in the presentation were located on the various sides of my body. |

Figure 3.

*Frequency of responses for Virtual Sickness Symptoms items.*


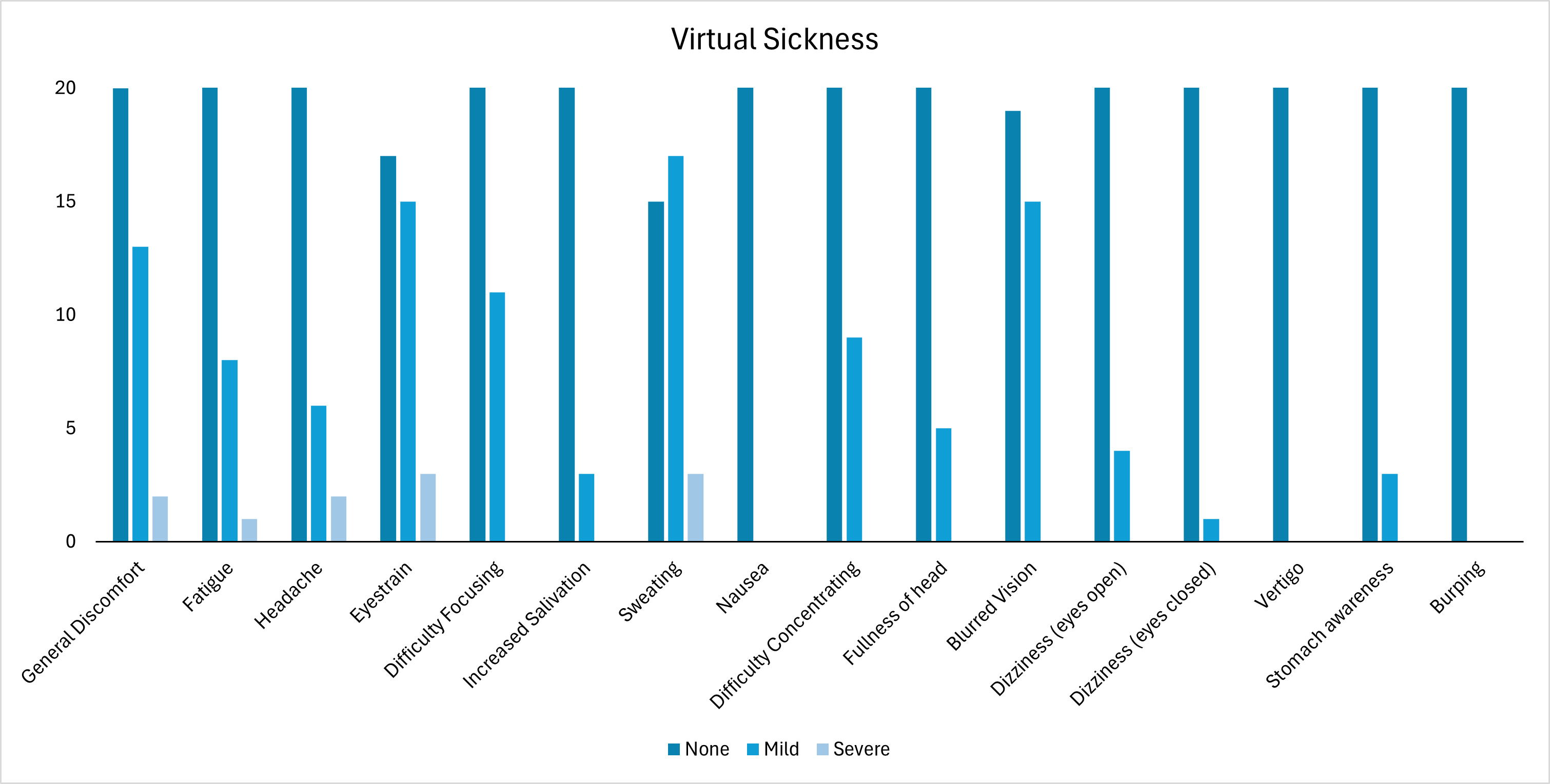

Supplement: Supplementary file 1 — Supplementary file1 (DOCX 207 KB) [file 13428_2025_2750_MOESM1_ESM.docx]
